# Impacts of sodium butyrate on intestinal mucosal barrier and intestinal microbial community in a weaned piglet model

**DOI:** 10.3389/fmicb.2022.1041885

**Published:** 2023-01-12

**Authors:** Han Liu, Jing Zhao, Wenju Zhang, Cunxi Nie

**Affiliations:** College of Animal Science and Technology, Shihezi University, Shihezi, China

**Keywords:** sodium butyrate, weaned piglet model, intestinal barrier function, intestinal bacteria community, growth performance

## Abstract

**Objective:**

Butyrate is thought to enhance intestinal mucosal homeostasis, but the detailed mechanism remains unclear. Therefore, further investigation on the mechanism of butyrate regulation of intestinal mucosal homeostasis was performed.

**Materials and methods:**

This study used weaned piglets with similar intestinal metabolic function to humans as a research model. The dietary supplemented 0.2% sodium butyrate group (0.2% S) and negative control group (CON) were established to detect the effects of butyrate on growth performance, intestinal tissue morphology, mucosal barrier function, and intestinal microbial community structure in weaned piglets.

**Results:**

There was an increase in average daily gain (ADG) during three different experimental periods and a reduction in average daily feed intake (ADFI) and feed-to-gain ratio (F:G) during days 1–35 and days 15–35 in 0.2% S compared with CON (*P* > 0.05). Furthermore, villus height in the ileum and duodenum was increased, and crypt depths in the colon and jejunum were reduced in both groups (*P* < 0.05). Moreover, the ratio of villus height and crypt depth (V/C) in 0.2% S both in the ileum and jejunum was significantly increased (*P* < 0.05) compared with CON. The relative mRNA expression of *PKC*, *MUC1*, *CLDN1*, and *ITGB1* was upregulated in the ileum of 0.2% S compared with CON (*P* < 0.05). The digesta samples of 0.2% S, both in the ileum (*P* < 0.05) and colon, contained greater intestinal bacterial abundance and diversity of probiotics, including *Lactobacillus*, *Streptococcus*, *Megasphaera*, and *Blautia*, which promoted amino acid metabolism and energy production and conversion in the colon and the synthesis of carbon-containing biomolecules in the ileum.

**Conclusion:**

In summary, dietary supplementation with 0.2% sodium butyrate was shown to have a tendency to improve the growth performance of weaned piglets and enhance intestinal mucosal barrier function *via* altering the gut microbiota.

## Introduction

Gut homeostasis in humans enhances nutrient absorption and utilization, fights against the invasion of pathogens, and also contributes to several human body systems (Elmassry et al., 2022). Increasing evidence is revealing that the interplay among endogenous or exogenous substances involved in intestinal metabolic function, gut microbiota, and the intestinal barrier system are key contributors to gut homeostasis in the host. Disturbance of intestinal homeostasis can result in a serious decline in host health, involving intestinal inflammatory diseases or intestinal nutrition and metabolic disorders. Intestinal barrier dysfunction can even cause changes in intestinal epithelial permeability and result in mucosal inflammation, including inflammatory bowel diseases (IBDs) (Laukoetter et al., 2008). Many endogenous or exogenous substances, such as short-chain fatty acids, are able to improve intestinal barrier function and change the microbial community structure in the intestinal tract. Exogenous functional short-chain fatty acids can be added to the diet at appropriate concentrations. Endogenous short-chain fatty acids originate from intestinal anaerobic fermentation and can reach luminal concentrations of 130 mM in the human gastrointestinal tract (Lozupone et al., 2012).

Butyric acid is an SCFA that participates in several metabolic processes of the host and has been widely studied regarding its ability to improve intestinal function. Butyric acid research regarding improving intestinal neoplasia, inflammatory bowel disease, and intestinal malabsorptive states is ever-deepening and holds promise for potential clinical therapy (Salvi and Cowles, 2021). Butyrate is also the main energy source for colonocytes, with at least 95% of luminally derived butyrate being utilized and absorbed in the colon (Donohoe et al., 2011; den Besten et al., 2013). Furthermore, butyrate activates SCFA-specific G protein-coupled receptors and regulates gene expression by hypoxia-inducible factor (HIF) stabilization and histone deacetylase (HDAC) inhibition to alter the mucosal environment (Davie, 2003; Kelly et al., 2015). This type of influence on the mucosal environment inhibits some pathogenic bacteria, including *Salmonella* (Rivera-Chávez et al., 2016). Moreover, butyrate may regulate the IL-10 receptor, zonulin, claudins, and occludin to reduce epithelial permeability and reinforce tight junctions and transepithelial resistance *in vitro* (Wang et al., 2012; Zheng et al., 2017). MUC2 is the primary mucin glycoprotein in the colon produced by goblet cells; MUC2 protein has been increased when treated with butyrate both *in vitro* and in human colonic biopsies (Hamer et al., 2008). Butyrate enhances epithelial barrier function by modulating antimicrobial peptide secretions, such as LL-37 (Raqib et al., 2006), RegIIIγ, and β-defensins (Zhao et al., 2018), in the gut epithelium. In addition, butyrate promotes the proliferation of certain beneficial bacteria in the intestine, such as *Bifidobacterium* and *Lactobacillus*; some of these beneficial bacteria further stimulate the production of SCFAs, and the beneficial bacteria also improve gut microbial barrier function (Liu et al., 2021).

Butyrate enhances host intestinal homeostasis and nutrient metabolism and is a potential research target in the treatment of human intestinal inflammation and nutrient absorption disorders. It is helpful that pigs have a similar gastrointestinal tract anatomy and physiology to humans, making them a useful research model (Lunney et al., 2021). We, therefore, performed a study involving the feeding of weaned piglets with sodium butyrate at a concentration of 0.2%. By investigating the effects of 0.2% sodium butyrate on growth performance, intestinal mucosal barrier function, and changes in intestinal microbial communities in the piglet model, the mechanism of sodium butyrate optimization of intestinal function was further clarified, and a theory for the use of sodium butyrate as a potential food additive in the future was proposed.

## Materials and methods

### Experimental design and animal treatment

A total of 60 crossbred piglets (Duroc × Landrace × Large White) with an average initial body weight (BW) of 5.8 ± 0.5 kg were weaned at 27 ± 1 days of age and then randomly divided into two groups with six replicates and five piglets per replicate. One group received a corn-soybean meal basal diet (CON), and the other received a corn-soybean meal basal diet supplemented with 0.2% sodium butyrate (0.2% S). The sodium butyrate used in all experiments (>99% purity) was obtained from Shanghai Aladdin Biochemical Technology Co., Ltd. (Shanghai, China). Feed and water were available *ad libitum* throughout the experiment. The basal diet was formulated to meet the nutrient requirements recommended by the NRC in 2012. The ingredient composition and nutrient content of the basal diet are given in Table 1. Diets were mixed and ground to pass through a 0.15-mm sieve. Dry matter, gross energy, calcium and total phosphorus, crude protein, ether extract, and ingredient contents of the basal diets were calculated. Piglet BW and feed intake were measured individually at the start, 14th day, and end of the experimental period and used to calculate the average daily feed intake (ADFI), average daily gain (ADG), and feed-to-gain ratio (F:G). All procedures used in the current experiments were approved by the Animal Care and Use Committee of Shihezi University (Shihezi, China).

**TABLE 1:** The ingredient composition and nutrient content of diets (%, as-fed basis).

Ingredient	Content (%)	Nutrient levels^b^	
Extruded maize meal	54.19	Gross energy (MJ/kg)	16.95
Dehulled soybean meal	20.70	Dry matter (%)	91.41
Extruded soybean	11.00	Crude protein (%)	20.26
Whey power	4.00	Ether extract (%)	8.11
Fish meal	3.00	Calcium (%)	0.87
Wheat bran	1.50	Total phosphorus (%)	0.71
Dicalcium phosphate	2.20		
Glucose	1.00		
Limestone	0.80		
L-Lysine⋅HCl	0.35		
L-Threonine	0.18		
DL-Methionine	0.05		
Tryptophan	0.03		
Vitamin-mineral premix^a^	1.00		
Total	100		

^a^Vitamin-mineral premix supplied per kg diet: vitamin A, 9,000 IU; vitamin D3, 3,000 IU; vitamin E, 20 IU; vitamin K3, 3 mg; vitamin B12, 0.2 mg; niacin, 30 mg; pantothenic acid, 15.0 mg; choline chloride, 400 mg; Zn, 75 mg; Mn, 60 mg; Fe, 75 mg; Cu, 150 mg; I, 0.35 mg; Se, 0.30 mg.

^b^Nutrient levels are calculated values.

### Sample collection

At the end of the feeding trial, six piglets (one pig per pen) of similar BW from each group were slaughtered after being fasted overnight, and samples of the duodenum, jejunum, ileum, and colon were taken through a sterile laparotomy and placed in neutral formalin for histological analysis or collected in centrifuge tubes and then immediately placed in liquid nitrogen and stored at −80°C for analysis of mRNA expression in ileal tissue. Briefly, ∼1.5 cm of the middle parts of the duodenum, jejunum, ileum, and colon were collected. Digesta from the ileum and the colon were obtained using centrifuge tubes, immediately placed in liquid nitrogen, and then stored at −80°C for analysis of the bacterial community.

### Intestinal morphology analysis

Samples from the ileum, colon, duodenum, and jejunum were embedded in paraffin and cut into 5-μm-thick sections. Six non-successive sections of each sample were stained with hematoxylin and eosin. Six well-oriented villi and their associated crypts per section were collected from each sample. The crypt depth and villus height of the ileum, colon, duodenum, and jejunum were measured and analyzed using a Leica Image Processing with Analysis System (Leica Imaging Systems Limited, Berlin, Germany).

### RNA extraction and quantitative real-time PCR analysis

Total RNA of the ileum was extracted with TRIzol reagent (Invitrogen, Carlsbad, CA, USA) after tissue homogenization and mixed with DNase I (Invitrogen). The obtained total RNA of INC (ileum negative control) and IS (ileum with 0.2% sodium butyrate) were examined using 1% agarose gel electrophoresis and a 2100 Bioanalyzer RNA Nanochip (Agilent, Palo Alto, CA, USA). Reverse transcription of total RNA was performed using a PrimeScript™ RT Reagent Kit (Takara, Dalian, China). Expression levels of β*-actin*, *claudin-1* (*CLDN1*), *mucin-1* (*MUC1*), *occludin* (*OCLD*), β*1 integrin* (*ITGB1*), *collagen* (*COL*), and *protein kinase C* (*PKC*) in ileal tissues were analyzed by a Roche LightCyler 480 system (Roche, Basel, Switzerland). The primer sequences for these genes are shown in Table 2.

**TABLE 2:** Primers used for quantitative real-time PCR (RT-PCR).

Gene	Forward (5′-3′)	Reverse (5′-3′)	Products (bp)
β-actin	ACACGGTGCCCATCTACGAG	GCTTCTCCTTGATGTCCCGC	165
Occludin	CTTTCTCAGCCAGCGTATTC	AGGCAAGCGTGGAGGCAACA	131
Mucin-1	CGGAAGCAGGCACCTATAAC	CAGAATACAGACCAGCACCA	131
β1 integrins	TAAGAGTGCCGTGACAACCG	TTCAGAACCTGCCCATAGCG	154
Collagen	TGCTGCTGCTATTGTCCTTG	ACTGTGCCTTGGTGTTGGAT	105
Protein kinase C	CTCACTGCCACAACACAACT	GCACGAGCGGTTCTTCACTG	124
Claudin-1	CATTGCTATCTTTGCCTGTG	GCCATAACCGTAGCCATAAC	151

The real-time PCR (RT-PCR) system was as follows: 10 μl of 2 × SYBR^®^ premix Ex Taq™ II, 0.5 μl of each forward and reverse primer (10 μmol/L), 2 μl of complementary DNA template, and 7.0 μl of double distilled water. The PCR reaction included an inactivation step at 95°C for 5 min, and this was followed by 35 cycles of denaturation at 95°C for 10 s, annealing at 60°C for 10 s, and extension at 72°C for 15 s. Each reaction was conducted in 20 μl volumes using a LightCycler 480 SYBR Green 1 Master (Roche, Basel, Switzerland). Each gene was performed with triplicate biological replicates and technical replicates. Results were calculated and represented using the 2^–ΔΔCT^ method.

### DNA extraction and 16S rRNA sequencing of intestinal microbes

Using a DNA Stool Mini Kit (Qiagen, Hilden, Germany), the ileal and colonic total DNA of digesta samples were isolated. The extracted DNA was checked using 1% agarose gel electrophoresis and a NanoDrop 2000 spectrophotometer (Thermo Fisher Scientific, Waltham, MA, USA). The quantified DNA was then stored at −20°C for further analysis. The V3–V4 regions of the bacterial 16S rRNA gene were amplified using TransStart^®^ Fastpfu DNA Polymerase (Takara, Dalian, China). The upstream primer and the downstream primer were 5′-barcode-ACTCCTACGGGAGGCAGCA-3′ and 5′-GGACTACHVGGGTWTCTAAT-3′, respectively. Amplification PCR was performed in a 20-μl reaction system: 10 ng template DNA, 1 U FastPfu polymerase, 1 × FastPfu buffer, 250 μM dNTP, and 0.1 μM each primer. PCR reaction conditions: 95°C for 2 min, 95°C for 30 s, 55°C for 30 s, 72°C for 30 s for 30 cycles, and then 72°C for 5 min. PCR products were first purified using an AxyPrep DNA Purification Kit (Axygen Biosciences, Union City, CA, USA) after being run through 2% agarose gel electrophoresis. On agarose gels, the PCR products were quantified by a QuantiFluor-ST Fluorimeter (Promega, Wisconsin, USA) using a PicoGreen dsDNA Quantitation Kit (Invitrogen, Carlsbad, CA, USA). Purified amplicons were gathered in equimolar ratios for 2 × 300 bp sequencing by Illumina MiSeq in Shanghai Majorbio Bio-pharm Technology Co., Ltd. (Shanghai, China) based on the standard protocols. Each treatment group had six replications.

### Bioinformatics analysis

QIIME (v.1.9.1) and Fastp (v.0.19.6) were used for quality control and sequence filtering with the following criteria: (1) sequencing reads were clipped with an average quality score of <20; (2) reads shorter than 50 bp were dropped; (3) reads with two nucleotides of mismatch in primer sequences or ambiguous nucleotides were deleted; and (4) paired reads with <10 bp overlap were discarded. Operational taxonomic units (OTUs) with a 97% identity cutoff were gathered by Uparse^1^ (v.7.0.1090), and the analysis of taxonomy of OTUs was performed using the Silva^2^ (Release132) 16S rRNA database. The α-diversity indices, including Chao and Shannon, were analyzed using Mothur v.1.30.2. PCoA tools in R language were used for principal coordinates analysis (PCoA). The histogram of linear discriminant analysis (LDA) distribution was implemented using LDA effect size analysis (LEfSe) software. The 16S rRNA gene sequencing information was analyzed by PICRUSt to predict biological functions (EggNOG database^3^) and metabolic pathways (KEGG database^4^) of the bacterial community of ileal and colonic content samples of weaned piglets.

### Statistical analysis

Data (growth performance, qRT-PCR, and intestinal morphology) were analyzed using the unpaired *t*-test in SPSS, version 19.0 (IBM Corporation, Armonk, NY, USA). Results are represented as means ± SEM. Data (α-diversity indices, predictive analysis of metabolic functions, and metabolic pathways) analyses were performed using the Wilcoxon rank-sum test. LDA analysis was performed using the non-parametric factorial Kruskal–Wallis (KW) sum-rank test. A *P*-value of <0.05 was considered to be statistically significant.

## Results

### Growth performance

According to these results, there were no significant differences in the ADG, ADFI, and F:G (*P* > 0.05) of weaned piglets during the different experimental periods (Table 3). However, 0.2% S showed a tendency to improve the ADG during three different experimental periods, as well as the F:G and ADFI during days 15–35 and days 1–35.

**TABLE 3:** Effects of dietary supplement with 0.2% sodium butyrate on growth performance in weaned piglet model.^a^

Item	CON	0.2% S	*P*-value
1–14 days			
ADG	227 ± 28.3	234.9 ± 15.8	0.13
ADFI	493.5 ± 30.7	517.3 ± 22.6	0.12
F:G	2.17 ± 0.1	2.2 ± 0.2	0.11
15–35 days			
ADG	478 ± 28.9	481.7 ± 13.9	0.15
ADFI	1057.9 ± 30.5	1025.7 ± 30.7	0.18
F:G	2.22 ± 0.1	2.12 ± 0.1	0.25
1–35 days			
ADG	377.5 ± 16.9	384.8 ± 20.9	0.08
ADFI	832.4 ± 25.8	820 ± 30.5	0.11
F:G	2.21 ± 0.2	2.12 ± 0.3	0.18

^a^Data were shown as the mean ± SEM (n = 30). CON, basic diet control; 0.2% S, basic diet with 0.2% sodium butyrate group; ADFI, average daily feed intake; ADG, average daily gain; F/G, feed-to-gain ratio.

### Intestinal mucosal morphology

**FIGURE 1:**
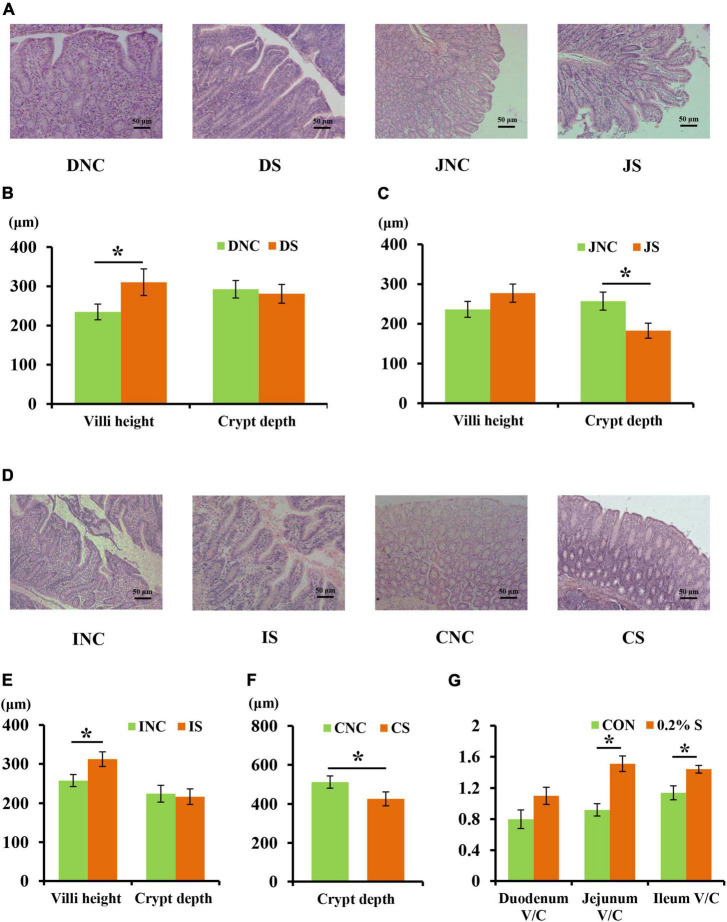
Effects of 0.2% sodium butyrate on the small intestine and colon mucosal morphology. **(A)** Mucosal morphology of duodenum and jejunum from weaned piglets. **(B)** The changes of villus height and crypt depth of duodenum. **(C)** The changes of villus height and crypt depth of jejunum. **(D)** Mucosal morphology of the ileum and the colon from weaned piglets. **(E)** The changes of villus height and crypt depth of ileum. **(F)** The changes of crypt depth of colon. **(G)** The ratio of the villus height and crypt depth (V/C) of the duodenum, the jejunum, and the ileum. Values are means ± SEM, *n* = 6. *P < 0.05. DNC, duodenum negative control; DS, duodenum with 0.2% sodium butyrate; JNC, jejunum negative control; JS, jejunum with 0.2% sodium butyrate; INC, ileum negative control; IS, ileum with 0.2% sodium butyrate; CNC, colon negative control; CS, colon with 0.2% sodium butyrate.

The mucosal morphology of the ileum, colon, duodenum, and jejunum are shown in Figure 1. The villus heights of IS (ileum with 0.2% sodium butyrate) and DS (duodenum with 0.2% sodium butyrate) were significantly higher than those in INC (ileum negative control) and DNC (duodenum negative control), respectively (*P* < 0.05) (Figures 1B, E). Conversely, the crypt depths in JS (jejunum with 0.2% sodium butyrate) and CS (colon with 0.2% sodium butyrate) were significantly lower than those in JNC (jejunum negative control) and CNC (colon negative control), respectively (*P* < 0.05) (Figures 1C, F). The ratio of the villus height and crypt depth (V/C) in 0.2% S was significantly higher compared with that in CON for both the jejunum and ileum (*P* < 0.05) (Figure 1G).

### Intestinal barrier function

**FIGURE 2:**
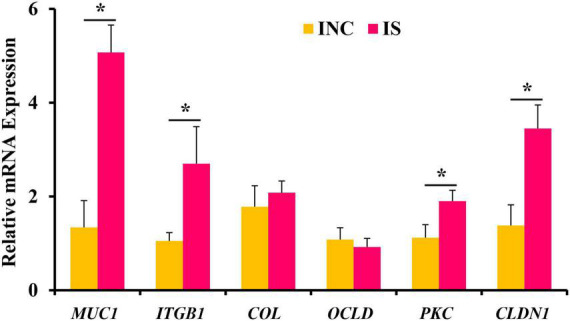
Effects of 0.2% sodium butyrate on the relative mRNA expressions of the ileal barrier functional gene. Bate-actin was used as an internal standard for normalization. Values are means ± SEM for three independent biological and technical replications, *n* = 6. *P < 0.05. CLDN1, claudin-1; MUC1, mucin-1; OCLD, occludin; ITGB1, β1 integrin; COL, collagen; PKC, protein kinase C.

The relative mRNA expressions of ileal barrier function-related genes are shown in Figure 2. Dietary supplementation with 0.2% sodium butyrate upregulated the relative mRNA expression of *CLDN1*, *MUC1*, *PKC*, and *ITGB1* in the ileum of the weaned piglet model (*P* < 0.05) (Figure 2). However, the relative mRNA expressions of *OCLD* and *COL* showed no significant difference between the INC and IS (*P* > 0.05).

### Bacterial community of digesta from the ileum and the colon

**FIGURE 3:**
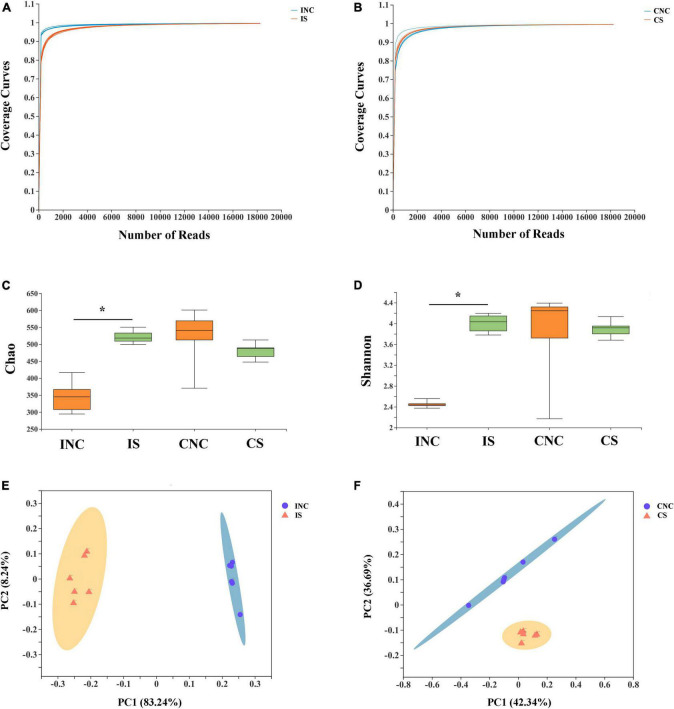
Alpha diversity and PCoA of bacterial communities between control and 0.2% sodium butyrate treatments in the ileum and the colon from weaned piglets. Sequencing coverage curves of **(A)** negative control (INC) and basal diet with 0.2% sodium butyrate (IS) from the ileum and **(B)** negative control (CNC) and basal diet with 0.2% sodium butyrate (CS) from the colon. **(C)** Chao index of the bacterial community and **(D)** Shannon index of the bacterial community in four groups. The PCoA analyses of bacterial communities between the control and sodium butyrate treatment group from **(E)** ileum and **(F)** colon. The results were analyzed by Wilcoxon rank-sum test, and *P < 0.05.

After quality control of 16S rRNA gene sequencing, a total of 470,445 and 432,056 clean reads were obtained at 0.2% S and CON, respectively (Table 4). The OTU numbers of IS, INC, CS, and CNC with six biological replications are shown in Table 4. The bioinformatics analysis was further performed according to OTU information. The coverage curves displayed a flat trend with the increasing number of sequencing reads, indicating that the sequencing reads in this experiment were sufficient to reveal the bacterial diversity of content samples from the colon and ileum (Figures 3A, B). Furthermore, the Chao index and Shannon index in IS were higher than those in INC, respectively (*P* < 0.05), while there were no significant differences (Figures 3C, D) in these indexes between the CNC and CS. PCoA analysis showed a clear differentiation between CON and 0.2% S in both the bacterial community structure of the ileum and the colon (Figures 3E, F), indicating that the addition of sodium butyrate changed the bacterial community structure in the ileum and the colon.

**TABLE 4:** Statistics of bacterial 16S rRNA gene amplicon sequencing for ileal and colonic content.^a^

Group ID	Clean reads	Average length (bp)	OTUs
INC1	48,572	424.62	186
INC2	61,439	413.79	250
INC3	25,883	416.95	233
INC4	47,961	415.08	248
INC5	34,086	417.09	217
INC6	34,817	414.54	251
CNC1	36,927	420.27	399
CNC2	21,440	417.53	508
CNC3	45,335	415.20	466
CNC4	34,634	417.17	483
CNC5	54,390	416.75	465
CNC6	24,961	425.23	266
IS1	30,571	438.81	452
IS2	35,442	440.75	415
IS3	34,083	436.31	470
IS4	33,890	436.50	439
IS5	34,317	438.22	429
IS6	38,135	440.01	407
CS1	35,636	435.76	399
CS2	37,744	439.04	380
CS3	42,076	437.79	369
CS4	40,878	437.52	409
CS5	30,800	436.61	390
CS6	38,484	436.98	395

^a^INC, ileum negative control; CNC, colon negative control; IS, ileum with 0.2% sodium butyrate; CS, colon with 0.2% sodium butyrate. Sequences with similarity scores ≥ 0.97 were clustered into an OTU.

**FIGURE 4:**
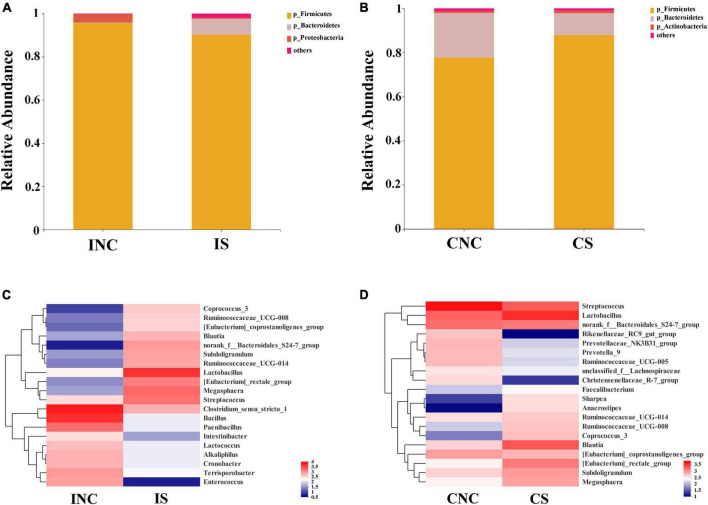
Bacterial diversity at the phylum and genus level in the ileum and the colon from weaned piglets of the control and treatment group. **(A)** Bacterial community bar plot of INC and IS at the phylum level in the ileum. **(B)** Bacterial community bar plot of CNC and CS at the phylum level in the colon. **(C)** Bacterial community bar plot of INC and IS at the genus level in the ileum. **(D)** Bacterial community bar plot of CNC and CS at the genus level in colon. INC, ileum negative control; CNC, colon negative control; IS, ileum with 0.2% sodium butyrate; CS, colon with 0.2% sodium butyrate.

At the phylum level, the relative abundance of *Firmicutes* in CS was enhanced compared to CNC (77.56% vs. 87.98%), and the relative abundance of *Firmicutes* in IS was reduced (95.61 vs. 90.25%) (Figure 4). The relative abundance of *Bacteroidetes* in IS (0.22 vs. 7.4%) was enhanced, while the *Proteobacteria* in IS (3.97 vs. 0.44%) was reduced compared to that in INC, respectively (Figure 4A). The relative abundance of *Bacteroidetes* in CS (20.29 vs. 9.84%) was reduced, whereas the *Actinobacteria* in CS (0.8 vs. 1.05%) was slightly enhanced (Figure 4B) compared to that in CNC, respectively.

At the genera level, the relative abundance of the top 20 bacterial communities (Figures 4C, D) in IS, INC, CS, and CNC are displayed in the heatmap. The relative abundance of dominant genera in INC, such as *Clostridium_sensu_stricto_1*, *Bacillus*, *Paenibacillus*, and *Terrisporobacter*, were decreased in IS (36.76 vs. 4.10%; 27.31 vs. 0.88%; 10.09 vs. 0.88%; and 5.13 vs. 1.10%), and the dominant genera in IS changed to *Lactobacillus* (30.04%), *Megasphaera* (12.95%), *Streptococcus* (10.90%), and *[Eubacterium]_rectale_group* (8.82%), which was richer than that in INC (Figure 4C). In the colon, the dominant genera including *Streptococcus* (36.47%), *Lactobacillus* (12.41%), *norank_f_Bacteroidales_S24-7_group* (10.73%), and *[Eubacterium]_coprostanoligenes_group* (5.66%) in CNC changed to *Lactobacillus* (23.58%), *Blautia* (13.27%), *Streptococcus* (12.55%), and *norank_f_Bacteroidales_S24-7_group* (8.34%) in CS (Figure 4D). In addition, the relative abundance of *[Eubacterium]_rectale_group*, *Anaerostipes*, *Sharpea*, and *Coprococcus_3* dramatically increased, but the *Prevotellaceae_NK3B31_group*, *Christensenellaceae_R-7_group*, and *Rikenellaceae_RC9_gut_group* were reduced in CS in comparison to CNC (Figure 4D).

**FIGURE 5:**
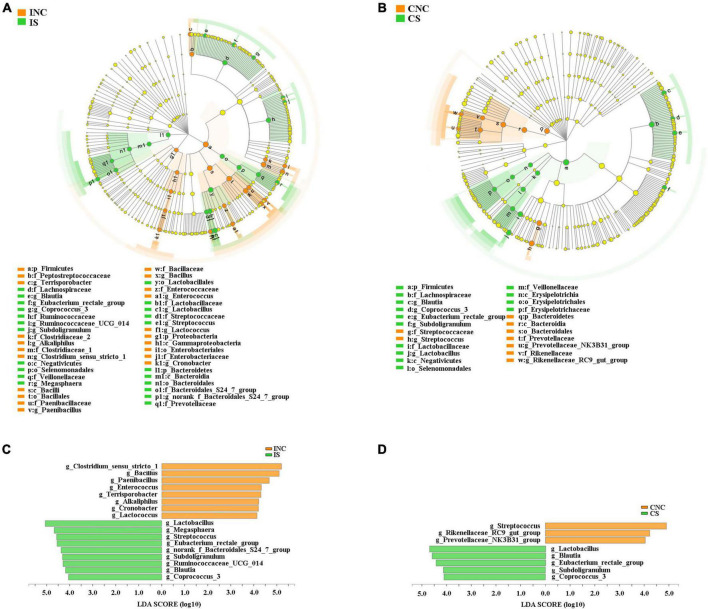
Linear discriminant analysis (LDA) effect size analysis (LEfSe) analysis showed significantly changed (*P* < 0.01) in bacteria between the control and treatment groups in the ileum and the colon. **(A)** Significantly changed bacteria between INC and IS from phylum to genus levels. **(B)** Significantly changed bacteria between CNC and CS from phylum to genus levels. **(C)** LDA bar chart showed significantly changed bacteria at genus level between INC and IS. **(D)** LDA bar chart showed significantly changed bacteria at genus level between CNC and CS. LDA score > 4 as the cutoff value. INC, ileum negative control; CNC, colon negative control; IS, ileum with 0.2% sodium butyrate; CS, colon with 0.2% sodium butyrate.

All differential bacteria of the ileum and the colon were demonstrated (Figures 5A, B) from the phylum to species level in cladograms of LEfSe between CON and 0.2% S. At the phylum level, the relative abundance of *Firmicutes* and *Proteobacteria* in the ileum and *Bacteroidetes* in the colon were significantly lower in 0.2% S than in CON (*P* < 0.05); while the relative abundance of *Bacteroidetes* in the ileum and *Firmicutes* in the colon were significantly higher in 0.2% S than in CON (*P* < 0.05) (Figures 5A, B). At the genera level, the relative abundance of *Clostridium_sensu_stricto_1*, *Bacillus*, *Paenibacillus*, *Enterococcus*, *Terrisporobacter*, *Alkaliphilus*, *Cronobacter*, and *Lactococcus* were among the top eight in INC (LDA SCORE > 4.0), while the relative abundance of *Lactobacillus*, *Megasphaera*, *Streptococcus*, *Eubacterium_rectale_group*, *norank_f_Bacteroidales_S24_7_group*, *Subdoligranulum*, *Ruminococcaceae_UCG_014*, *Blautia*, and *Coprococcus_3* were signally boosted in IS instead of INC (*P* < 0.05) (Figure 5C). Furthermore, the relative abundance of *Rikenellaceae_RC9_gut_group*, *Streptococcus*, and *Prevotellaceae_NK3B31_group* were highest in CNC (LDA SCORE > 4.0), but the relative abundance of *Lactobacillus*, *Blautia*, *Eubacterium_rectale_group*, *Subdoligranulum*, and *Coprococcus_3* were notably enhanced in CS and not in CNC (*P* < 0.05) (Figure 5D).

### Predicted functional profiles of microbial communities using PICRUSt

**FIGURE 6:**
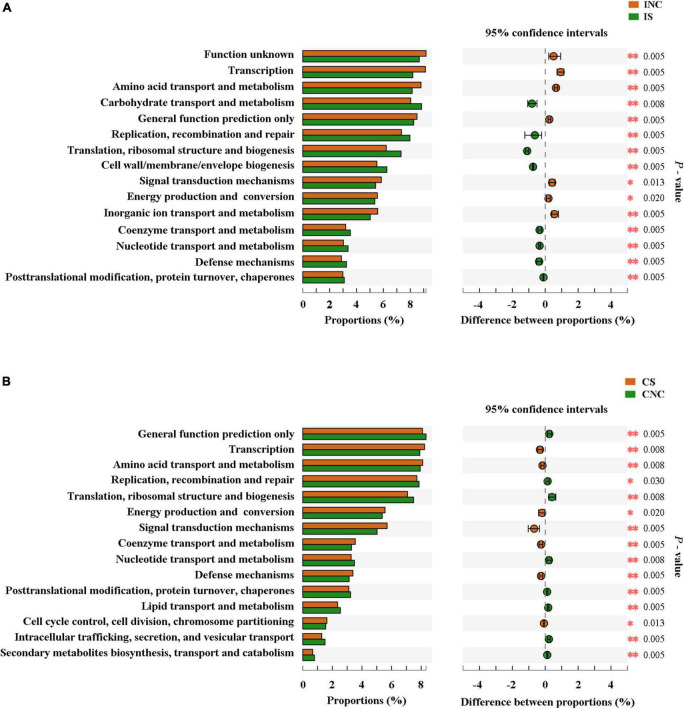
Comparison of the bacterial predicted biological functions (COG level 1) between the control and treatment group from the ileum and the colon using PICRUSt. **(A)** COG function prediction analysis of ileal bacteria between INC and IS. **(B)** COG function prediction analysis of colonic bacteria between CNC and CS. INC, ileum negative control; CNC, colon negative control; IS, ileum with 0.2% sodium butyrate; CS, colon with 0.2% sodium butyrate. *P < 0.05 and ^**^P < 0.01.

**FIGURE 7:**
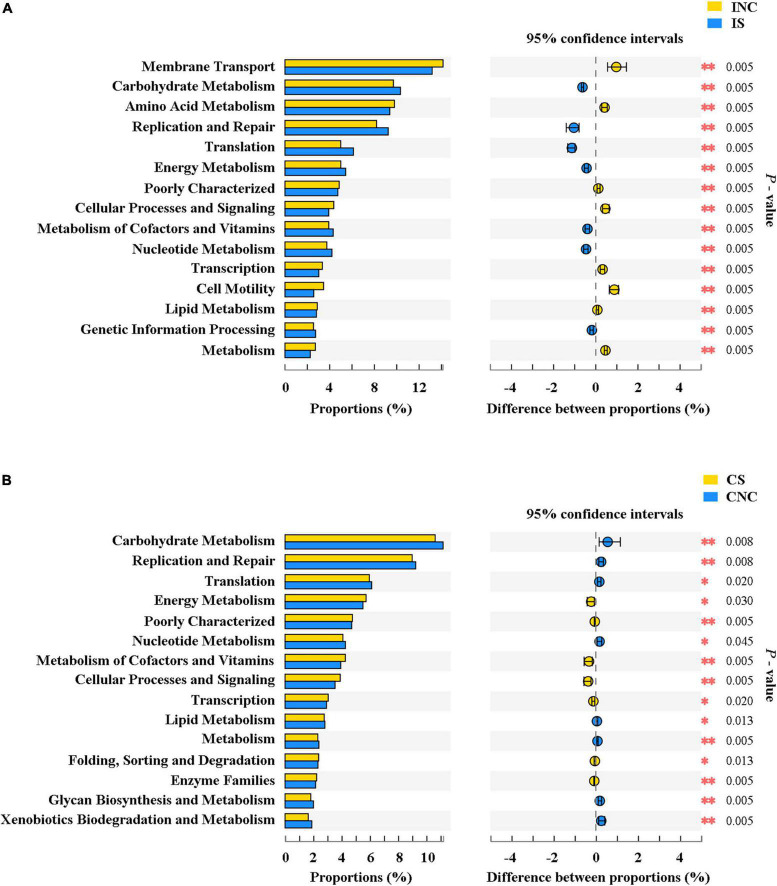
The mean proportion and significant difference in predicted metabolism pathways (KEGG) between the control and treatment group from the ileum and the colon using PICRUSt. **(A)** KEGG pathways analysis of ileal bacteria between INC and IS. **(B)** KEGG pathways analysis of colonic bacteria between CNC and CS. INC, ileum negative control; CNC, colon negative control; IS, ileum with 0.2% sodium butyrate; CS, colon with 0.2% sodium butyrate. *P < 0.05 and ^**^P < 0.01.

**FIGURE 8:**
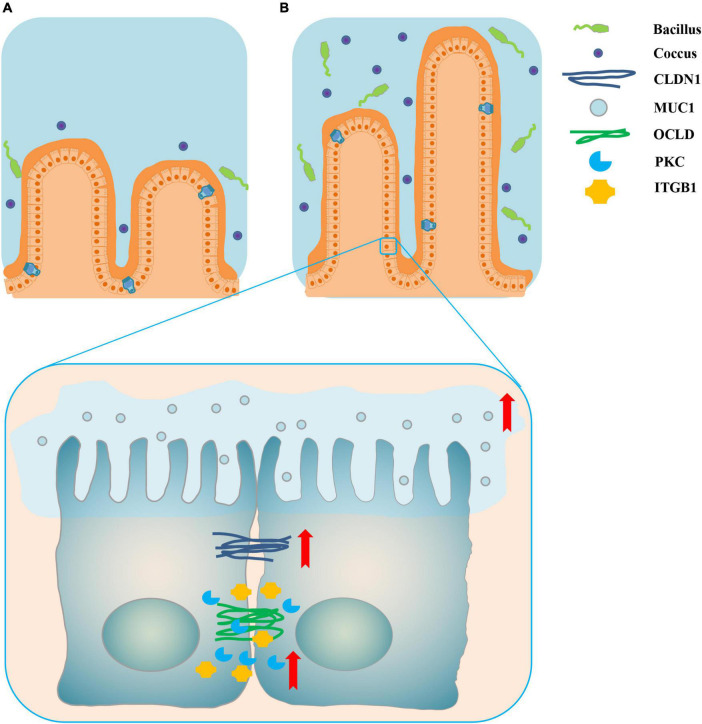
Schematic representation of dietary supplementation of 0.2% sodium butyrate improves ileal mucosal barrier function in piglets. **(A)** The ileal mucosal barrier status in the INC group. **(B)** The promotion of ileal mucosal barrier function by sodium butyrate in IS. The red arrows indicate that the relative mRNA expressions of ileal barrier functional proteins are significantly upregulated. Dietary supplementation of 0.2% sodium butyrate significantly increased the villus height of the ileum, upregulated the relative mRNA expressions of the ileal barrier functional gene, and increased the relative abundance of some probiotics such as *Lactobacillus*, *Streptococcus*, and *Blautia*.

The top 15 proportions (%), notably different predicted biological functions (COG level 1), and the KEGG pathways of bacteria in digesta from the ileum and the colon with and without sodium butyrate are displayed in Figures 6, 7. According to these results, bacteria with replication, recombination, and repair; carbohydrate transport and metabolism; translation; ribosomal structure and biogenesis; cell wall/membrane/envelope biogenesis; and coenzyme transport and metabolism functions were significantly more enriched in IS than in INC (*P* < 0.01) (Figure 6A). The predicted metabolic pathways involved in microorganisms, such as replication and repair, carbohydrate metabolism, translation, energy metabolism, metabolism of cofactors and vitamins, and nucleotide metabolism, were significantly enriched in IS compared to INC (*P* < 0.01) (Figure 7A). The significantly increased levels of bacteria after dietary supplementation of 0.2% sodium butyrate in the colon improved the functions of transcription, amino acid transport and metabolism, energy production and conversion, signal transduction mechanisms, and coenzyme transport and metabolism (*P* < 0.01) (Figure 6B). In CS, energy metabolism, poorly characterized, metabolism of cofactors and vitamins, cellular processes and signaling, and transcription were significantly enriched (Figure 7B) (*P* < 0.01). In summary, dietary supplementation of 0.2% sodium butyrate improved mucosal barrier function and the structure of the bacterial community of the ileum (Figure 8).

## Discussion

The effects of butyrate on the host are embodied in such aspects as growth performance, intestinal nutrition metabolism, and intestinal microbial community structure. With pigs as a research model, studies into the ability of butyrate to improve growth performance, nutrient metabolism, and the microbial community structure of the intestines have become more in-depth and systematic with the help of new and efficient detection technologies (Mazzoni et al., 2008; Le Gall et al., 2009). Based on the measurements on the 15th and 35th days in this study, it was found that the ADG was higher in 0.2% S than CON in the three different experimental periods; in the last 20 days and throughout the experimental period, the ADFI and F:G of 0.2% S was lower than that of CON. These results indicated that sodium butyrate showed a trend toward improving the growth performance of weaned piglets, but the difference between these groups was not significant. These results were consistent with previous studies investigating dietary supplementation with different concentrations of sodium butyrate (Biagi et al., 2007; Chen et al., 2019a). However, dietary supplementation with sodium butyrate and organic acids (such as benzoic acid) (Wei et al., 2021) or coated sodium butyrate (Upadhaya et al., 2020) have been shown to improve growth performance. In addition, dietary supplementation with sodium butyrate also reduced obesity from high-fat diets and contributed to reducing weight gain (Liu et al., 2021). The concentration and form of sodium butyrate seem to be critical in regulating growth performance, and the proper concentration plays a role in achieving the expected growth target.

To further clarify the mechanism of dietary sodium butyrate on animal intestinal nutrition metabolism, the intestinal mucosal morphology of piglets in different treatment groups was detected. According to the present results, the villus heights of DS and IS were significantly higher than those in DNC and INC, respectively; the crypt depths of JS and CS were significantly lower than those in JNC and CNC, respectively; and the V/C in 0.2% S was also significantly higher than those in CON for both the jejunum and ileum. Increased intestinal villi height decreases the inflammatory response and intestinal epithelial permeability, weakens dysfunction of intestinal motor function, and even increases the intestinal absorption area (Collins, 2001). Furthermore, crypts play a role in the generation and transportation of intestinal epithelial cells; decreased intestinal crypt height causes an increase in the rate of mature cells (Clevers, 2013). Therefore, dietary sodium butyrate has been shown to improve intestinal mucosal barrier function in piglets, and this is consistent with the results of previous studies on the improvement of animal intestinal barrier function by endogenous butyrate (Martin-Gallausiaux et al., 2021). This improvement was mainly achieved by regulating the oxygen content of the colon, epithelial permeability, mucosal barrier function-related proteins, the thickness of the intestinal mucus layer, and promoting the secretion of antimicrobial peptides from the intestinal epithelium. Exogenous oral infusion of butyric acids (11 mM) also tended to increase villi height in the ileum of a pig model (Zhou et al., 2020).

To deeply investigate the effects of sodium butyrate on intestinal barrier function, the relative mRNA expression of intestinal mucosal barrier function-related genes (*CLDN1*, *MUC1*, *ITGB1*, *OCLD*, *COL*, and *PKC*) in the ileum was checked by qRT-PCR. The relative mRNA expression of *CLDN1*, *MUC1*, *PKC*, and *ITGB1* was upregulated in 0.2% S compared with CON. Mucins mainly play roles in maintaining the structure and biological function of the intestinal mucosa and regulating the structure of the intestinal microbial community (McGuckin et al., 2011). PKC regulates occludin phosphorylation and promotes the assembly of epithelial tight junctions (Jain et al., 2011). CLDN1, which is a member of the claudin family of transmembrane proteins, is a major component of tight junction strands (Monaco et al., 2021). PKC and CLDN1 are both mainly involved in maintaining the structure and function of tight junctions. Integrins, including ITGB1, regulate the assembly of adhesive junctions in the intestinal tract and are then mainly involved in the rapid renewal and digestive functions of the intestinal tract (Breau et al., 2009). Therefore, dietary supplementation of sodium butyrate significantly regulated the expression of genes related to the intestinal mucosal barrier and improved intestinal mucosal barrier function in this study.

Intestinal bacteria are important participants in intestinal nutrient metabolism and play important roles in nutrient absorption, mucosal barrier homeostasis, immunomodulation, and defense against pathogens in pigs (Huang et al., 2015). Based on the above results that diets supplemented with sodium butyrate improved intestinal barrier function, the effects of sodium butyrate on intestinal bacteria also need to be investigated. In the present study, the Chao index and the Shannon index of the ileum were more greatly increased in 0.2% S compared with CON, indicating that the addition of 0.2% sodium butyrate to the feed promoted the growth of ileal bacteria and improved the diversity of the microbial community in weaned piglets. The bacterial diversity reflects the health and stability of the gut microbial community, which is beneficial to the host (Salonen et al., 2012). Moreover, the results of PCoA analyses demonstrated that there were significant differences in the microbial community composition between CON and 0.2% S, indicating that the composition of the gut microbiota was changed by the sodium butyrate in weaned piglets.

At the phylum level, the relative abundance of *Proteobacteria* from the ileum in 0.2% S decreased when compared with CON in this study. The relative abundance of *Proteobacteria* in a balanced gut-associated microbial community is usually small (Eckburg et al., 2005). A recent study shows that an increase in *Proteobacteria* could be a potential diagnostic microbial signature of epithelial dysfunction as well as dysbiosis in the intestinal microbiota (Shin et al., 2015). Moreover, the relative abundance of *Firmicutes* was increased in the colon after supplementing 0.2% sodium butyrate. *Firmicutes* is the dominant phylum in the intestinal microbiota of piglets. The expansion of *Firmicutes* is understood to enhance the body’s capacity for energy acquisition from the diet, which might further improve the growth performance of weaned piglets (Turnbaugh et al., 2006).

At the genera level, the relative abundances of *Lactobacillus*, *Streptococcus*, *Megasphaera*, and *Blautia* in the intestinal tract were increased following supplementation with sodium butyrate, which agrees with previous findings (Wei et al., 2021). *Lactobacillus* enhances human and animal health and is considered a probiotic (Dunne et al., 2001). Moreover, *Lactobacillus* is considered to be involved in the production of butyrate (Berni Canani et al., 2016), which is a known immunoregulatory factor (Iacob and Iacob, 2019) and was enhanced with the addition of sodium butyrate in this study. Furthermore, certain *Lactobacillus* species contribute to the improvement of intestinal barrier function and the exclusion of pathogens by upregulating the expression of tight junction proteins (Anderson et al., 2010) and mucin (Mack et al., 2003), which is consistent with the qRT-PCR results in this study. *Firmicutes* intestinal bacteria play important roles in carbohydrate metabolism (Walker et al., 2011). In addition, *Blautia* is involved in the acetate synthesis pathway in the colon (Louis et al., 2014). Increases in *Megasphaera* and *Blautia*, which belonged to *Firmicutes*, promote intestinal carbohydrate metabolism balance in a weaned piglet model. *Streptococcus* is considered to participate in the processes of amino acid utilization (Neis et al., 2015) and the production of short-chain fatty acids (Corr et al., 2009), and short-chain fatty acids contribute to maintaining intestinal homeostasis (Iacob and Iacob, 2019). In addition, D-alanine (Miyauchi et al., 2012) and exopolysaccharide (Chen et al., 2019b), produced by *Streptococcus thermophilus*, and yogurt fermented by *Streptococcus thermophilus* 1131 (Usui et al., 2018) can enhance intestinal barrier mucosal function and mitigate intestinal inflammation. Moreover, the relative abundance of the *[Eubacterium]_rectale*_*group* in the ileum was decreased in a gut model of ulcerative colitis patients, indicating that *Eubacterium rectale* might improve intestinal function through butyrate metabolism (Vermeiren et al., 2012). We also found that the number of gene tags involved in amino acid metabolism and energy production and conversion in the colon and the number of gene tags involved in the synthesis of carbon-containing biomolecules in the ileum were markedly improved in 0.2% S compared with CON. The results suggested that sodium butyrate supplementation might affect amino acid metabolism and carbohydrate metabolism by altering the gut microbiota; however, it is necessary to further clarify the relationship between carbohydrate and amino acid metabolism and sodium butyrate supplementation.

## Conclusion

Dietary supplementation of 0.2% sodium butyrate produced slight changes in the growth performance of a weaned piglet model. However, 0.2% sodium butyrate supplementation increased the relative mRNA expression of *MUC1*, *ITGB1*, *PKC*, and *CLDN1* of the ileum, improved intestinal mucosal morphology, and ultimately enhanced intestinal mucosal barrier function by altering the gut microbiota, including increasing *Lactobacillus*, *Streptococcus*, *Megasphaera*, and *Blautia*. These findings contribute to a better understanding of how sodium butyrate modulates gut health through nutrient interaction with microorganisms and provides a basis for the clinical application of sodium butyrate in regulating intestinal microbes.

## Data availability statement

The data presented in the study are deposited in the NCBI repository, accession number PRJNA831435.

## Ethics statement

This animal study was reviewed and approved by the Animal Care and Use Committee of Shihezi University (Shihezi, China).

## Author contributions

CN and WZ conceived and designed the research. HL and JZ conducted the research. HL wrote the manuscript and analyzed the data. JZ and CN wrote a part of the manuscript and assisted in the analysis of data. CN and HL critically reviewed the manuscript and contributed to the language review. All authors read and approved the final manuscript.

## Footnotes

1
http://drive5.com/uparse/

2
http://www.arb-silva.de

3
http://eggnog.embl.de/

4
http://www.genome.jp/kegg
